# Downregulation of CYP39A1 Serves as a Novel Biomarker in Hepatocellular Carcinoma with Worse Clinical Outcome

**DOI:** 10.1155/2021/5175581

**Published:** 2021-12-31

**Authors:** Dan Li, Tao Yu, Junjie Hu, Jie Wu, Shi Feng, Qingxue Xu, Hua Zhu, Xu Zhang, Yonggang Zhang, BenHong Zhou, Lijuan Gu, Zhi Zeng

**Affiliations:** ^1^Department of Pharmacy, Renmin Hospital of Wuhan University, Wuhan, China; ^2^Department of Oncology, Integrated Traditional Chinese and Western Medicine, The Central Hospital of Wuhan, Tongji Medical College, Huazhong University of Science and Technology, Wuhan, China; ^3^College of Pharmacy, Hubei University of Chinese Medicine, Wuhan, China; ^4^Central Laboratory, Renmin Hospital of Wuhan University, Wuhan, China; ^5^Department of Pathology, Renmin Hospital of Wuhan University, Wuhan, China

## Abstract

**Background:**

CYP39A1 is a poorly characterized metabolic enzyme that has been investigated in a few tumors. However, the role of CYP39A1 in hepatocellular carcinoma (HCC) has not yet been clarified. In this study, the expression and clinical significance of CYP39A1 in HCC were explored.

**Methods:**

CYP39A1 protein expression was detected in Akt/c-Met-induced HCC mice and 14 paired fresh HCC samples as well as another 159 HCC and matched noncancerous tissues. Meanwhile, the mRNA expression was analyzed by GEO and TCGA analysis and validated in 14 paired fresh HCC tissues. Furthermore, the relationships between CYP39A1 expression and clinicopathologic features as well as prognosis were analyzed. HCC cell growth changes were analyzed by cell viability assays after CYP39A1 overexpression and then validated after CYP39A1 knockout by DepMap database analysis.

**Results:**

CYP39A1 protein expression was lower expressed in HCC mouse models, and its mRNA and protein expression were also downregulated in HCC compared with noncancerous liver tissues. Higher CYP39A1 expression was associated with well differentiation. Moreover, survival analysis indicated that lower CYP39A1 expression was associated with poorer overall survival. In addition, HepG2 and SMMC-7721 cell viability were inhibited after CYP39A1 overexpression. Genome-wide CRISPR/Cas9 proliferation screening indicated that knockout of CYP39A1 could promote HCC cell growth. Likewise, p-NF-*κ*B and Nrf2 were suppressed after CYP39A1 overexpression. It is worth mentioning that total bile acid, total bilirubin, and direct bilirubin were significantly increased in the patients with low CYP39A1 expression.

**Conclusions:**

Downregulation of CYP39A1 is associated with HCC carcinogenesis, tumor differentiation, and poor overall survival, suggesting that CYP39A1 may serve as a tumor suppressor gene and novel biomarker for HCC patients.

## 1. Introduction

Hepatocellular carcinoma (HCC) is one of the most common malignancies with increasing burden globally. Until recently, systemic therapies for HCC have been limited, and the prognosis for advanced HCC is generally poor [1]. It remains essential to identify new biomarkers for early diagnosis and prognostic evaluation in HCC.

Cytochrome P450 (CYP450) is a monooxygenase, which mainly exists in human liver and intestines. It can catalyze the metabolism of a variety of endogenous and exogenous substances, including approximately 90% of drugs in the clinic. Recently, the roles of CYP450 family members in tumor diagnosis and treatment have been gradually investigated in an increasing number of studies. For instance, CYP1A2 expression levels can serve as a biomarker of HCC recurrence caused by hepatitis C [2], and CYP3A5 can act as a tumor suppressor gene by regulating the mTORC2/Akt signaling pathway in HCC [3]. Furthermore, CYP2W1 can be used as an independent prognostic biomarker in liver cancer [4], and CYP17A1 can be used as a molecular marker in the diagnosis of liver cancer [5].

CYP39A1, as a novel member of the cytochrome P450 superfamily, is known as an oxysterol 7 alpha-hydroxylase that participates in the metabolism of 24 hydroxycholesterol (24OHC) [6–8]. It is located on human chromosome 6 and contains 14 coding exons. CYP39A1 is mainly expressed in the liver, and its mRNA was also discovered in nonpigmented epithelial cells in the eye and brain [9]. A recent whole-exome sequencing case-control study indicated that rare CYP39A1 variants were associated with exfoliation syndrome involving the anterior chamber of the eye [10]. Furthermore, the CYP39A1 single nucleotide polymorphism was associated with adverse reactions to chemotherapeutic drugs [11, 12]. In addition to its function in metabolism and inflammation reported in previous studies [13–15], the role of CYP39A1 in carcinoma has been gradually investigated. Currently, CYP39A1 is expressed at lower levels in malignant melanoma [16] and cholangiocarcinoma [17]. However, there have been no definitive data showing a correlation between CYP39A1 expression and the clinicopathological features of HCC. The exact role of CYP39A1 in the development and progression of HCC remains unclear.

Therefore, CYP39A1 mRNA and protein expression were analyzed by bioinformatic methods and validated in HCC and noncancerous tissue samples as well as HCC mouse models. Then, the correlations between CYP39A1 expression and clinicopathological factors, overall survival, and serum biochemical indices were investigated. Moreover, the function of CYP39A1 in HCC cell lines was further analyzed.

## 2. Materials and Methods

### 2.1. Tissue Sample and Data Collection

A total of 159 patients with HCC (123 men and 36 women; age range, 26-73 years) were included in the present study. All patients did not receive radiotherapy or chemotherapy before surgery. Demographic and clinical pathological data were collected including age, sex, tumor size, tumor differentiation, distant metastasis, nodal metastasis, and TNM stage. Additionally, serum biochemical indices were obtained from the inspection report of medical records. A total of 159 formalin-fixed, paraffin-embedded HCC tissues, and matched adjacent noncancerous liver tissue samples were selected. An additional 14 pairs of fresh samples of HCC and noncancerous liver tissues were gathered, snap-frozen immediately in liquid nitrogen, and stored at -80°C following surgery for real-time PCR and Western blot analysis. The research was approved by the Ethics Committee of Renmin Hospital of Wuhan University which conformed to the provisions of the Declaration of Helsinki.

Furthermore, 159 patients were followed up for survival analysis; however, 81 patients were lost to follow-up. The overall survival duration was from the date of operation to the date of death or the last known survival date. Moreover, 81 cases from the GEO database were also utilized for survival analysis as a complementary validation.

### 2.2. Immunohistochemistry (IHC)

IHC staining procedures were performed according to a standard protocol that was described in detail in our previous study [18]. In brief, the sections were deparaffinized with xylene and rehydrated with ethanol, then washed with deionized water and stirred. The sections were then treated with 3% H_2_O_2_ and then antigen retrieval by citric acid buffer (pH 6.0). After sealing at room temperature for 20 min with 5% bovine serum albumin, the slices are incubated overnight at 4°C with primary antibody (1 : 400 dilution for human liver tissues and 1 : 100 dilution for mice liver tissues, Abcam, USA). Then, sections were incubated with horseradish peroxidase-labeled polymer with secondary antibody (UltraSensitive™ SP (Mouse/Rabbit) IHC Kit-9710; Maixin Bio, Fujian, China) at room temperature for 15 min each. Then, the reaction products were stained with 3,3′-diaminobenzidine (DAB) and lightly counterstained with hematoxylin. The sections with PBS instead of primary antibody served as negative control.

### 2.3. Evaluation of IHC Staining

CYP39A1 expression levels were evaluated according to the average score of two independent pathologists' evaluations. If the difference of score was ≥2, the final score was determined by a third pathologist. CYP39A1 was mainly located in the cytoplasm and presented in a different staining intensity, hence, CYP39A1 expression in tumor cells was classified according to a four-tier grading system (scores: 0 = absent, 1 = weak, 2 = moderate, and 3 = strong staining), as described in a previous study [19]. Generally, a score less than or equal to 1 was considered as negative, and a score more than 1 was considered as positive.

### 2.4. Quantitative Real-Time Polymerase Chain Reaction (qRT-PCR)

Total RNA was extracted from fresh HCC and matched noncancerous liver tissue samples using TRIzol reagent (Invitrogen, USA) according to the manufacturer's instructions, and qRT-PCR analysis was carried out with Power SYBR Green (Takara, Japan). The primer sequences used were as follows: *β*-actin forward: AGCGCGCATCCCCCAAAGTT; *β*-actin reverse: GGGCACGAAGGCTCATCATT; CYP39A1 forward: ACAATGGACCTGAACAACT; and CYP39A1 reverse: AAGACACTCTGGCAACTG.

### 2.5. Western Blotting

The homogenized matched liver samples were extracted by RIPA and then quantified by BCA kit (Thermo, USA). Briefly, the supernatant of the lysate was used for Western blot analysis. The protein was separated by electrophoresis in a 10% SDS-PAGE and then transferred onto a PVDF membrane, and the membranes were blocked with 5% skim milk powder, then it was incubated with CYP39A1 (1 : 1000 dilution, ab129334, Abcam), Anti-Nrf2 (NF-E2 related factor 2, 1 : 1000 dilution, ab92946, Abcam), Keap 1 (1 : 1000 dilution, 7705, Cell Signaling Technology), or Phospho-NF-*κ*B p65 (1 : 1000 dilution, 3033, Cell Signaling Technology) at 4°C overnight. After incubation with the horseradish peroxidase (HRP) conjugated IgG antibody (1 : 5000 dilution, Sigma Chemical Co., St Louis, MO) at room temperature for 1 h, the chemiluminescence phototope-HRP kit (Pierce, Rockford, IL) was employed, and the band intensity was visualized by Quantity One software (BioRad, Hercules, CA). The CYP39A1 protein level was normalized to that of Vinculin (1 : 10000 dilution, ab129002, Abcam) in 14 fresh liver tissues or beta tubulin (1 : 1000 dilution, 2148S, Cell Signaling Technology) in the *in vitro* transfection study or HCC mouse models. These antibodies can be used both in human and mouse.

### 2.6. Hydrodynamic Transfection to Construct a Rapid HCC Model

Hydrodynamic transfection was performed in FVB/N mice (*n* = 6) from Charles River (Beijing, China) as described in our previous study [20] to construct a rapid HCC model. In brief, the three plasmids pT3-EF1*α*-HA-myr-AKT, pT3-EF1*α*-V5-c-Met, and pCMV-SB with the corresponding quality of 20 *μ*g, 20 *μ*g, and 1.6 *μ*g were diluted in a 2 mL saline (0.9% NaCl) solution and promptly injected into the lateral tail vein of the FVB/N mice within 7 s. Approximately 6 weeks later, the HCC model was successfully constructed. The mice were divided into four groups. Wild type groups and the other three AKT/c-Met induced HCC modeling groups. Low-dose (122 mg/kg/day) and high-dose (244 mg/kg/day) of osthole or vehicle were intraperitoneally injected once daily for 3 weeks after 3 weeks of hydrodynamic injection. At 6 weeks posthydrodynamic injection, the livers were taken after pentobarbital anesthesia. The animal study was reviewed and approved by the Animal Ethics Committees of the Hubei University of Chinese Medicine. CYP39A1 expression in the wild-type and model groups was detected by IHC, and the corresponding protein was determined by Western Blotting.

### 2.7. Lentiviral Plasmid Construction and Cell Proliferation Assay

To construct pLV-EGFP-flag-CYP39A1, a 1451 bp CYP39A1 gene fragment was successfully amplified and inserted into the GV492 plasmid with the restriction enzyme BamHI-AgeI (New England Biolabs, USA). Then, it was packaged into recombinant lentiviral particles after enzymatic digestion and DNA sequencing. HepG2 and SMMC-7721 cells in the exponential growth phase were infected with empty vector (EV) and CYP39A1 overexpression (OE) virus. To achieve an optimal infection state, 4 × 10^6^ lentivirus was added to 4 × 10^5^ cells per well in a six-well plate according to the MOI of HepG2 and SMMC-7721 cells.

For the cell viability assay, HepG2 and SMMC-7721 cells were infected with EV or OE for 24, 48, 72, 96, and 120 h, and then 2000 cells per well were plated into 96-well plates. After CCK-8 treatment for 4 hours, the variation in the absorption rate at 450 nm with time in the EV and OE groups was compared. The relative CYP39A1 mRNA and protein expression levels were detected.

### 2.8. Analysis of Publicly Available Data

Human HCC datasets of patients with HCC and corresponding clinical data, as well as their CYP39A1 mRNA expression data, were retrieved from the publicly available GEO database (http://www.ncbi.nlm.nih.gov/gds) and The Cancer Genome Atlas (TCGA) datasets (https://cancergenome.nih.gov/). Three independent datasets, GSE14520, GSE45267, and GSE64041, were used to calculate the relative expression level of CYP39A1 mRNA between noncancer livers and HCC. Then, the correlation between CYP39A1 mRNA expression and clinicopathologic features from the TCGA database (https://xenabrowser.net/) was utilized for further validation. Moreover, survival analysis was validated by analyzing the data from GSE54236.

In order to analyze the Genome-wide CRISPR/Cas9 Proliferation Screening Data in HCC Cell Lines, Gene knockout data for 808 cell lines of 29 primary diseases from CRISPR were obtained from the DepMap database (https://depmap.org/portal/), and then 22 HCC cell lines (HLF, JHH4, SNU398, HUH7, JHH2, HUH6, SNU387, SKHEP1, JHH6, HEPG2, JHH7, LI7, SNU182, SNU886, SNU761, SNU449, PLCPRF5, SNU423, SNU475, JHH1, HUH1, and JHH5) were employed to extract CYP39A1 knockout data. As described in a previous study [21], the CERES dependency score was used to represent the changes in cell growth after genes of interest were knocked out. A score of 0 indicates that a gene is not essential for cell growth; correspondingly, a score of -1 is comparable to the median of all pan-essential genes [22]. The lower the score, the more likely the gene is an oncogene. The higher the score, the more likely the gene is a tumor suppressor gene.

### 2.9. Statistical Analysis

Statistical tests were carried out by SPSS 20.0. Student's *t*-test was used to analyze the difference between 2 groups. One-way analysis of variance was employed to compare the data more than 2 groups. The chi-square test was used to analyze the relationship between CYP39A1 expression and the clinicopathological factors of HCC. For survival analysis, the Kaplan-Meier method was employed to draw the survival curves and the difference was compared by log-rank test. *P* values less than or equal to 0.05 were considered to be statistically significant.

## 3. Results

### 3.1. Expression of CYP39A1 Protein in HCC Mouse Model

**Figure 1:**
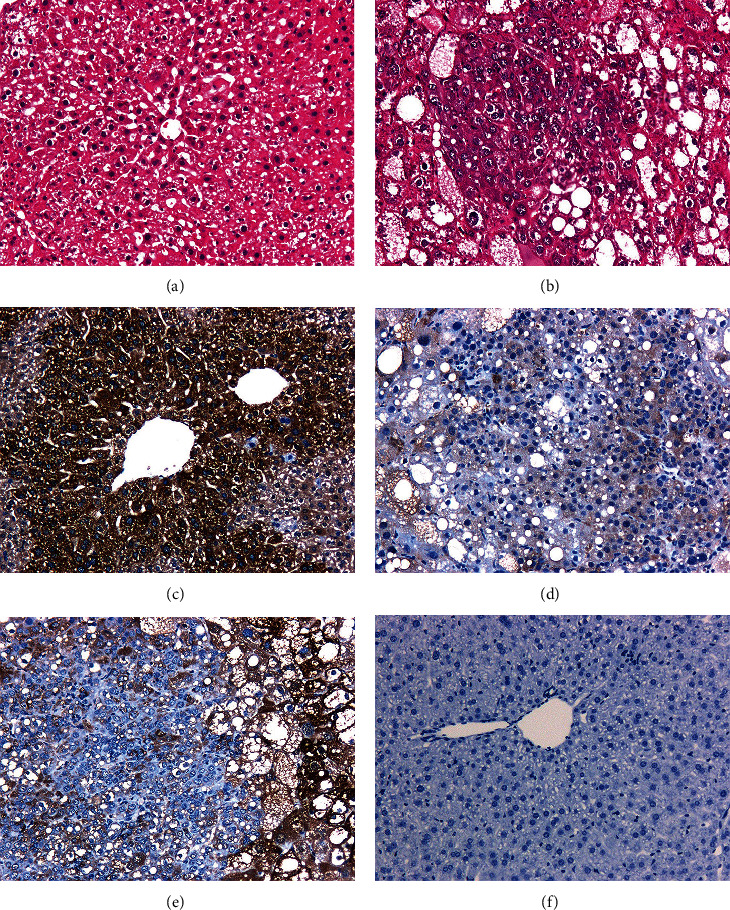
H&E and IHC staining of CYP39A1 proteins in representative tissue of HCC mice. (a) Wild-type mice, H&E staining; (b) HCC mice, H&E staining; (c) wild-type mice, IHC staining of CYP39A1; (d) HCC mice, IHC staining of CYP39A1; (e) the junction of hepatocellular carcinoma and paracancerous liver tissue in the same field of HCC mice, IHC staining of CYP39A1; (f) negative control; original magnification, ×100.

The results of H&E staining and histological analysis confirmed that the HCC mouse model was successfully established. Representative tissue specimens showed that the hepatic lobule structure was clear and that the hepatic cell cord was well-organized in the WT mice. In the Akt/c-Met-induced HCC mouse model, the tumor cells showed hepatocytic differentiation by morphology. Meanwhile, HCC cells were arranged in solid or nest-like growth patterns, showing loss of normal hepatic architecture, such as reduction or loss of the normal reticulin framework. Cytological atypia was very obvious, such as an increased nucleus-to-cytoplasm ratio (Figures 1(a) and 1(b)). Next, CYP39A1 protein expression in Akt/c-Met-induced HCC mouse model was assessed by performing IHC. The results indicated that the immunoreaction for CYP39A1 protein was more intense in the normal liver tissues of WT mice than in the cancer tissues of Akt/c-Met-induced HCC mice (Figures 1(c)–1(f)).

### 3.2. CYP39A1 mRNA Expression Was Downregulated in Human HCC

**Figure 2:**
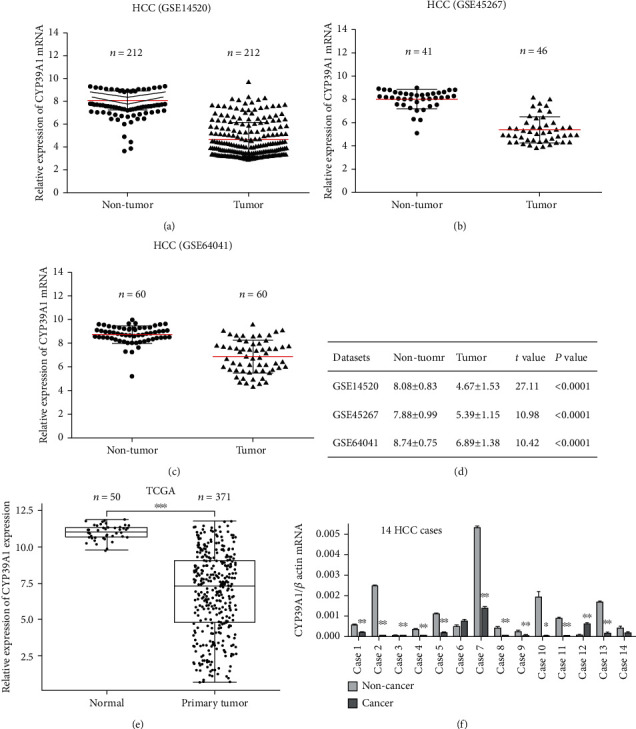
Differential mRNA expression of CYP39A1 between HCC and noncancerous liver tissues. (a) The GSE14520 dataset with two cohorts showed that CYP39A1 mRNA expression was decreased in HCC tissues compared with adjacent noncancerous liver tissues (*P* < 0.001, paired *t*-test). (b) The GSE45267 dataset showed that CYP39A1 mRNA expression was decreased in HCC tissues compared with unpaired noncancerous liver tissues (*P* < 0.001, unpaired *t*-test). (c) Sixty paired HCC biopsies from an unselected patient population with all tumor stages from GSE64041 indicated that CYP39A1 mRNA was lower in HCC tissues than in adjacent noncancerous liver tissues (*P* < 0.001, paired *t*-test). (d) Relative expression value of CYP39A1 in HCC and noncancerous liver tissues in the three GSE datasets. (e) CYP39A1 mRNA expression was downregulated in HCC tissues in the TCGA dataset analysis. (f) CYP39A1 mRNA expression in 14 fresh-frozen liver tissues. (C: cancer tissue; N: noncancerous tissue).

The distinction of CYP39A1 mRNA expression between noncancerous liver and HCC tissues was analyzed from three independent microarray datasets, namely, GSE14520, GSE45267, and GSE64041. Overall, the three GSE datasets indicated that CYP39A1 mRNA expression was downregulated in HCC compared with normal or adjacent noncancerous liver tissues (*P* < 0.0001, Figures 2(a)–2(d)). Moreover, CYP39A1 mRNA expression was lower in HCC tissues in the TCGA dataset analysis (*P* < 0.001, Figure 2(e)). To further verify the results of bioinformatics analysis, 14 fresh tissue samples were collected and detected by real-time PCR. Similarly, it was also found that CYP39A1 mRNA expression in the cancer tissues (7.14%, 1/14) was lower than that in matched adjacent noncancerous tissues (78.57%, 11/14) (*P* < 0.01, paired *t*-test, Figure 2(f)).

### 3.3. Expression and Location of CYP39A1 Protein in HCC Tissues and Adjacent Noncancerous Liver Tissues

**Figure 3:**
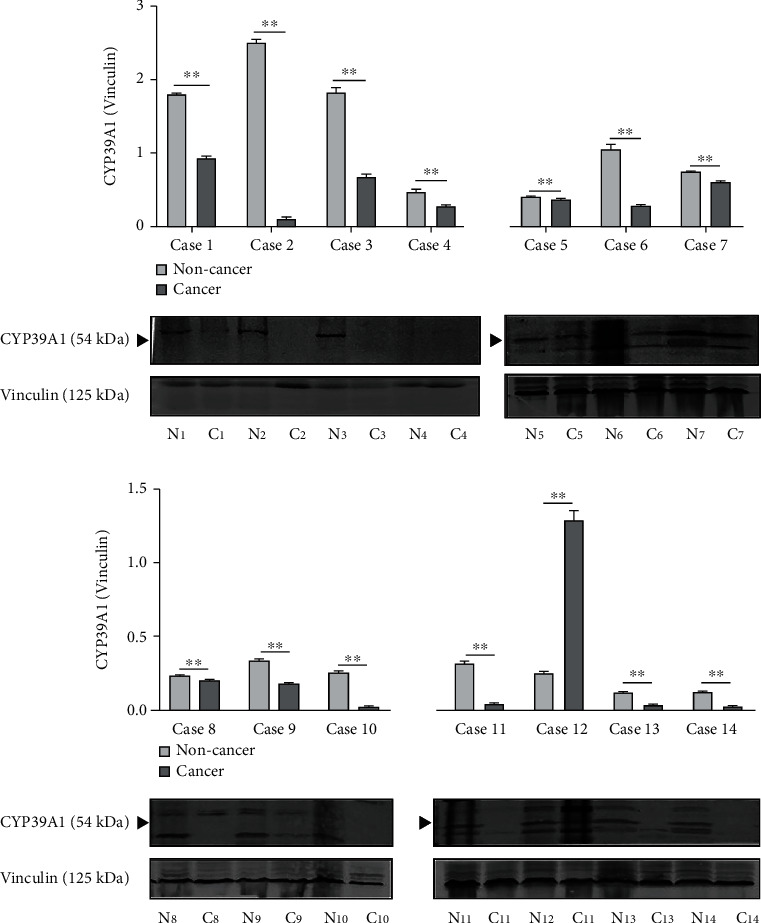
CYP39A1 protein expression was relatively lower in fresh-frozen HCC tissues than in adjacent noncancerous liver tissues. (C: cancer tissue; N: noncancerous tissue.)

Next, the above mentioned 14 paired HCC and noncancerous liver tissues were employed to compare the protein expression difference by Western blotting analysis. Study results indicated that CYP39A1 protein expression was also lower expressed in HCC tissues (7.14%, 1/14) than that in matched noncancerous liver tissues (85.71%, 12/14) in these paired liver samples (*P* < 0.01, paired *t*-test, Figure 3).

**Figure 4:**
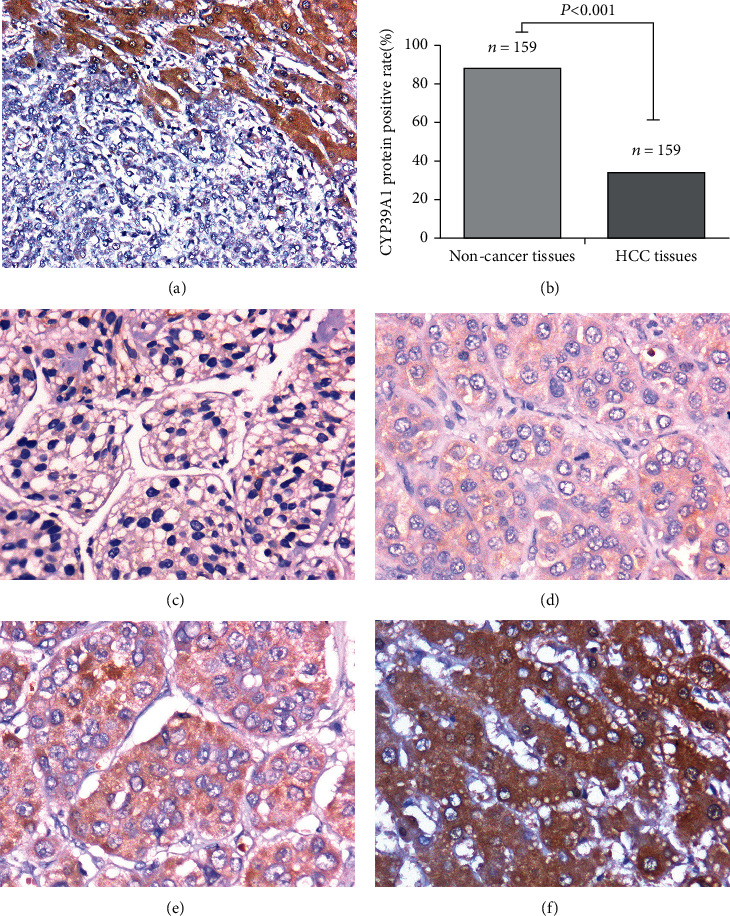
CYP39A1 protein expression in HCC and noncancerous liver tissues and its immunohistochemical staining in representative tissue specimens. (a) Immunohistochemical staining of CYP39A1 proteins in representative liver tissue specimens. Left bottom corner represents cancer tissues and top right corner represents noncancer tissues. DAB staining (brown); nuclear counterstaining (hematoxylin); original magnification, ×100; (b) CYP39A1 protein positive rate in adjacent noncancerous liver tissues and HCC tissues (*P* < 0.001, cancer vs. noncancerous tissues). Statistical analysis was performed by the chi-squared test; (c)–(e) Expression of CYP39A1 in representative tumor cells of HCC ((c) score = 0, (d) score = 1, and (e) score = 2); (f) Expression of CYP39A1 in representative normal hepatocytes ((f) score = 3). DAB staining (brown); nuclear counterstaining (hematoxylin); original magnification, ×200. Among them, (c) and (d) were defined as negative expression of CYP39A1, and (e) and (f) were defined as positive expression of CYP39A1.

To investigate the protein expression and location of CYP39A1 in noncancerous liver and HCC tissues, the CYP39A1 protein expression was detected further in a larger size of 159 HCC tissues and its adjacent noncancerous liver tissues by IHC staining. Our results indicated that CYP39A1 was mainly expressed in the cytoplasm of hepatocellular carcinoma cells or hepatocytes rather than in stromal cells. The immunoreaction for CYP39A1 protein was more intense in the normal cells than that in the HCC cells (Figure 4(a)). Similarly, it was also found that the positive expression rate of CYP39A1 was higher in normal liver tissues (88.05%) than that in HCC tissues (33.96%) (*P* < 0.001, Figure 4(b)). The scores of CYP39A1 protein expression ranging from 0, 1, and 2 in representative HCC tissue specimens were presented in Figures 4(c)–4(e). Moreover, a typical representative IHC image of noncancerous liver tissue (score = 3) was shown in Figure 4(f). Generally, a score less than or equal to 1 was considered as negative, and a score more than 1 was considered as positive.

### 3.4. Relationship between CYP39A1 Protein Expression and Clinicopathologic Features of HCC

**Figure 5:**
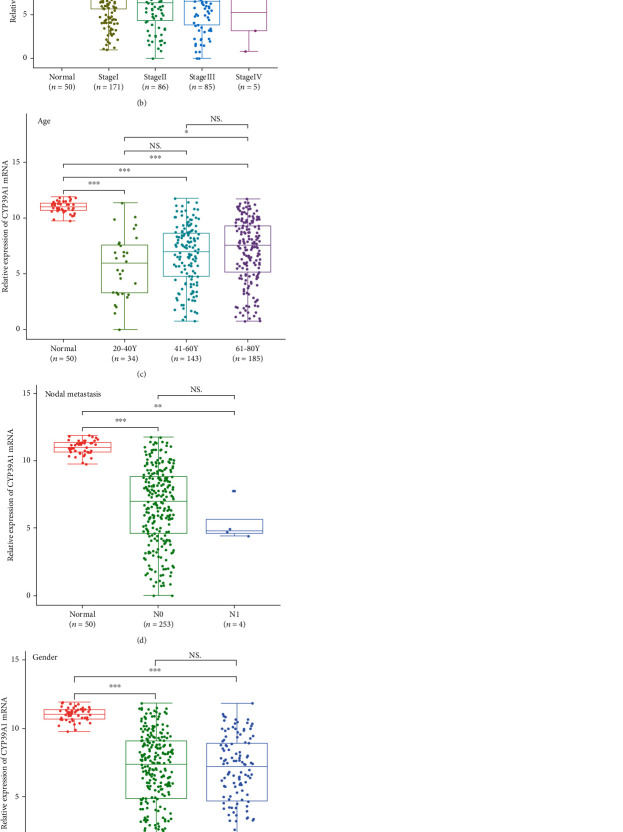
Correlation between CYP39A1 mRNA expression and clinicopathologic features of HCC in the TCGA database. (a) Tumor grade; (b) cancer stage; (c) patient age; (d) nodal metastasis; (e) gender; (f) histological type. (HCC: hepatocellular carcinoma; FCL: fibrolamellar carcinoma; mixed: hepatocholangio carcinoma).

The relationship between CYP39A1 protein expression detected by IHC and the clinicopathologic features of 159 HCC patients was further analyzed. The statistical results were shown in Table 1. The results indicated that CYP39A1 expression was positively correlated with tumor cell differentiation. High levels of CYP39A1 were associated with well-differentiated HCC tissues (*P* = 0.001). However, CYP39A1 expression was not correlated with sex, age, tumor size, nodal metastasis, distant metastasis, or TNM stage (*P* > 0.05; Table 1). Furthermore, bioinformatic analysis also indicated that CYP39A1 mRNA expression was correlated with tumor grade (cell differentiation). There was a significant difference among grade 1, grade 2, and grade 3 (*P* < 0.05; Figure 5(a)), whereas CYP39A1 mRNA expression was not correlated with cancer stage, age, nodal metastasis, gender, or histological type of HCC (*P* > 0.05; Figures 5(b)–5(f)).

### 3.5. Correlation between CYP39A1 Expression and HCC Patients' Overall Survival

**Figure 6:**
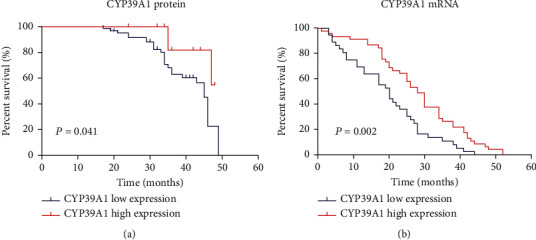
Relationship of CYP39A1 expression and patient prognosis. (a) Relationship of CYP39A1 protein expression and overall survival (*P* = 0.041) (log-rank test). IHC score = 0 or 1 was considered CYP39A1 protein negative expression, *n* = 61, and IHC score = 2 or 3 was considered CYP39A1 protein positive expression, *n* = 17; (b) GSE54236 showed the relationship of CYP39A1 mRNA expression and overall survival (*P* = 0.002). Low expression group, *n* = 36; high expression group, *n* = 45.

In order to further investigate the correlation between CYP39A1 expression and the prognosis of HCC patients, the overall survival information for 78 patients in our study was obtained by a postoperative follow-up. Kaplan-Meier analysis and the log-rank test showed that the expression of CYP39A1 protein was also associated with HCC overall survival (*P* = 0.041, Figure 6(a)). Furthermore, in order to verify the role of CYP39A1 in the prognosis of HCC patients, GSE54236, with 81 patients' survival information, was utilized to compare CYP39A1 mRNA expression and patients' overall survival. Our results indicated that CYP39A1 mRNA expression was positively correlated with HCC patients' survival (*P* = 0.002, Figure 6(b)). Therefore, our findings indicated that lower expression of CYP39A1 might serve as a prognostic marker in HCC patients with worse clinical outcomes.

### 3.6. CYP39A1 Inhibited the Proliferation of HCC Cells In Vitro

**Figure 7:**
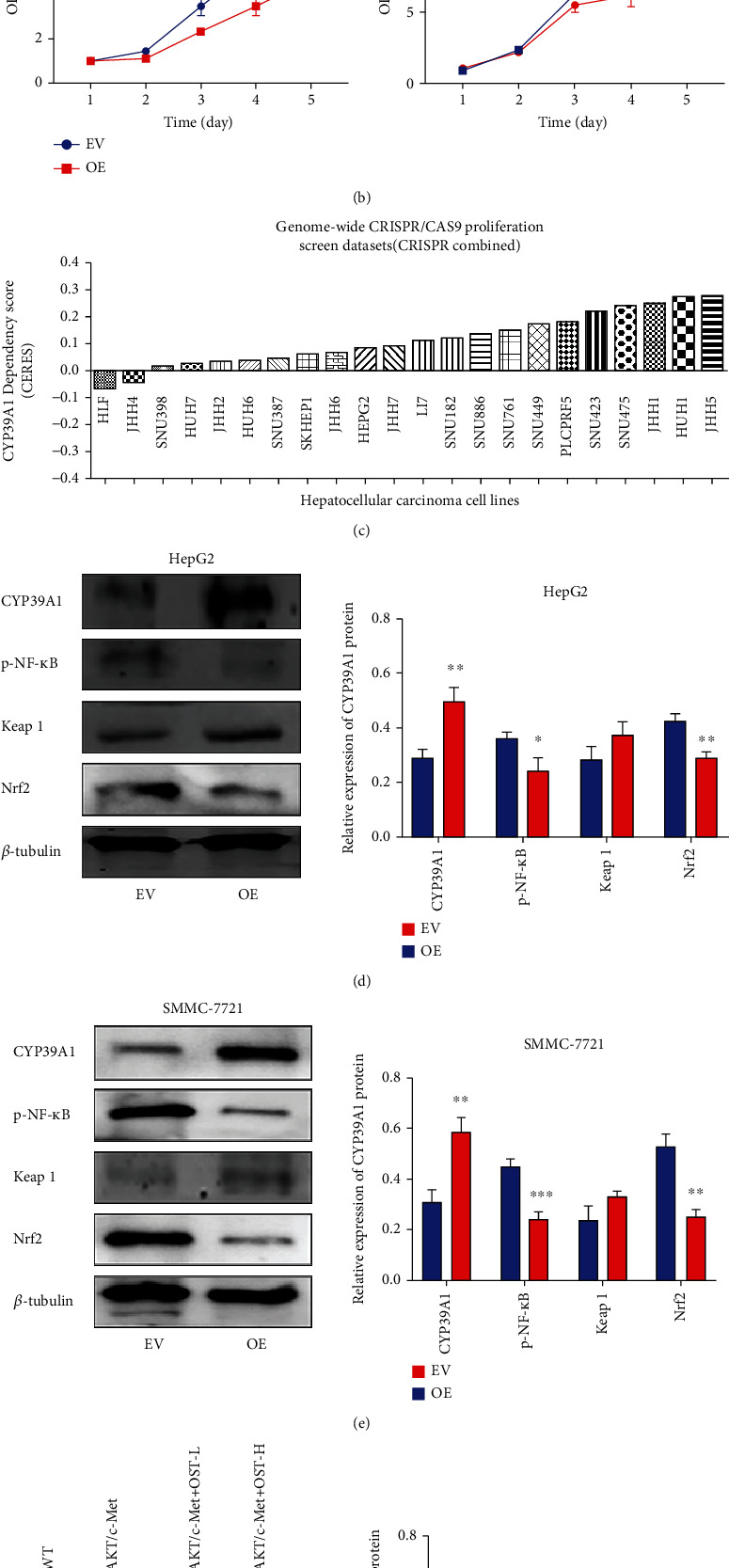
The relationship between CYP39A1 and the cell growth of HCC. (a) Relative CYP39A1 mRNA expression in the EV and OE groups after infection with CYP39A1 lentiviral plasmid in HepG2 and SMMC-7721 cells; (b) cell viability was inhibited after CYP39A1 was overexpressed in HepG2 and SMMC-7721 cells; (c) CERES dependency score of CYP39A1 in 22 HCC cell lines in the CRISPR combined database; (d) and (e) the relative protein expression of CYP39A1, p-NF-*κ*B, Keap1, and Nrf2 after infection with CYP39A1 lentiviral plasmid in HepG2 and SMMC-7721 cells; histograms indicated the relative expression levels of CYP39A1, p-NF-*κ*B, Keap1, and Nrf2. Data were expressed as the mean ± S.D.; *n* = 3. ^∗^*P* < 0.05, ^∗∗^*P* < 0.01, ^∗∗∗^*P* < 0.001 versus the EV group; (f) the protein expression of CYP39A1, p-NF-*κ*B, Keap1, and Nrf2 in AKT/c-Met-induced HCC mouse models were determined by Western blot analysis; histograms indicated the relative expression levels of CYP39A1, p-NF-*κ*B, Keap1, and Nrf2. Data were expressed as the mean ± S.D.; *n* = 3. ^#^*P* < 0.05, ^##^*P* < 0.01, ^###^*P* < 0.01 versus the WT group; ^∗^*P* < 0.05, ^∗∗^*P* < 0.01, ^∗∗∗^*P* < 0.001 versus the AKT/c-Met group.

The results from our clinical samples indicated that CYP39A1 was lower expressed in HCC and correlated with the differentiation and prognosis of HCC. Then, cell viability was detected in HepG2 and SMMC-7721 cells infected with the CYP39A1 empty vector (EV) or overexpressing (OE) lentiviral plasmid. CYP39A1 mRNA was overexpressed in the OE group compared with the EV group (*P* < 0.01, Figure 7(a)). Meanwhile, the proliferation of HepG2 and SMMC-7721 cells was inhibited after CYP39A1 overexpression (*P* < 0.05, Figure 7(b)). To further understand the function of CYP39A1 in more HCC cell lines, the DepMap database was employed to analyze cell growth after the genes of interest were knocked out. The CERES dependency scores of 22 HCC cell lines for CYP39A1 in the genome-wide CRISPR/Cas9 screening database were extracted. The score was more than 0 in most of the HCC cell lines (20/22) (Figure 7(c)). As it described in the materials and methods section, the higher the score, the more likely the gene is a tumor suppressor gene. Therefore, it can be inferred that CYP39A1 might serve as a tumor suppressor gene in HCC. In order to clarify the possible mechanism of CYP39A1 in HCC, the protein of HepG2 and SMMC-7721 cells infected with the CYP39A1 EV or OE lentiviral plasmid was collected and determined. Similarly, CYP39A1 protein expression was also significantly higher in the OE group while p-NF-*κ*B and Nrf2 protein were decreased after CYP39A1 protein was overexpressed (Figures 7(d) and 7(e)). Furthermore, we found that CYP39A1 was decreased in the tumor cells of Akt/c-Met-induced HCC mouse model (*P* < 0.001), however, it was increased in the oral administration of osthole group, especially in the high dose of osthole group. Likewise, the p-NF-*κ*B and Nrf2 were activated in the AKT/c-Met induced HCC models; however, with the increasing of CYP39A1 protein expression by treating with osthole, p-NF-*κ*B and Nrf2 protein levels were decreased in a dose-dependent manner. Moreover, Keap 1 protein was upregulated with the increased expression of CYP39A1 after treating with osthole (Figures 7(f)). Thereby, these all results suggested that CYP39A1 might be involved in the regulation of HCC growth through p-NF-*κ*B and Keap 1-Nrf2 pathway.

### 3.7. Correlation between CYP39A1 and Serum Biochemical Indices in HCC

To investigate whether the change in CYP39A1 will affect liver function and other serum biochemical indices, the serum biochemical indices of HCC patients, including alanine aminotransferase (ALT), aspartate aminotransferase (AST), total bile acid (TBA), total bilirubin (TBIL), direct bilirubin (DBIL), and total cholesterol (TCh), were detected. The relationships between CYP39A1 expression and serum biochemical indices were shown in Table 2. Interestingly, TBA, TBIL, and DBIL were significantly lower in the high CYP39A1 expression group than that in the low CYP39A1 expression group. Conversely, PA, UA, Cr, etc. were significantly higher in the high CYP39A1 expression group than that in the low CYP39A1 expression group.

## 4. Discussion

HCC, a multifactorial disease characterized by multistep-multistage hepatocarcinogenesis, is the third leading cause of cancer-related mortality [23]. The incidence and mortality of HCC are still increasing rapidly. Hence, it is urgent to develop specific strategies and effective treatments for HCC patients. It has been reported that gene dysregulation could induce the occurrence and development of numerous types of carcinomas [24], including HCC [24]. In this study, our findings indicated that CYP39A1 protein and mRNA levels were lower in HCC than in noncancerous liver tissues, suggesting that decreased CYP39A1 expression might be associated with HCC carcinogenesis. Similarly, this finding was consistent with a previous report that CYP3A4 was identified as a tumor suppressor gene related to a poor prognosis in HCC [25, 26].

Furthermore, for CYP2C subfamily members, CYP2C8, CYP2C9, and CYP2C19 were reported to be potential serum biomarkers for the early diagnosis of HCC, and high expression levels of them were associated with a reduced risk of death [27]. Also, downregulation of CYP2A6 and CYP2C8 in HCC tissues was linked to poor overall survival and recurrence-free survival [28]. These study results were consistent with our research results that CYP39A1 was downregulated in HCC. However, these studies have some limitations. First, most of the studies were only performed by bioinformatic analysis, which needs larger population studies for further validation. Second, CYP expression in most of these studies was limited to the mRNA level, which may not necessarily reflect the activities of CYPs. Third, further well-designed studies concentrating on functional and phenotypic validation are needed to clarify whether these genes can serve as novel markers of HCC. Recently, newly identified CYP family members, such as CYP4F2, CYP4F12, CYP4V2 [29], and CYP2W1 [30], have also shown correlations with prognosis in HCC.

At present, mouse was the commonly used animal model of HCC to evaluate the pathogenesis of HCC, with the advantages of small size and very close genomic sequence to human [31]. The commonly used HCC model was carcinogen-induced mice, tumor transplanted mice, and transgenic mice. However, there are some shortcomings of these models, such as long modeling cycle, high cost of use or inhomogenous performance among individuals, and these models could not meet the requirement of research on the interaction of multiple genes and multiple signaling pathways in the occurrence of liver cancer. The Akt/c-Met-induced HCC mice were launched by Chenxin's Lab that refers to Sleeping Beauty (SB) transposon-mediated high-pressure caudal vein hydrodynamic plasmid transfection [32], which has become one of the effective methods for the establishment of mouse liver cancer models because of the short modeling cycle, uniform tumor formation, and simple operation [32]. In this study, the Akt/c-met-induced HCC mice were successfully established as in our previous research [20] and used to evaluate the CYP39A1 differential expression in HCC occurrence which was consistent with the results in human HCC tissues. In addition, in order to study the possible mechanism of CYP39A1 in HCC, the related protein with HCC was detected. It was reported that NF-*κ*B and Nrf2 was activated in HCC [33–36], which was also found in our HCC model group with the downregulation of CYP39A1. However, CYP39A1 expression was increased after treating with osthole, while p-NF-*κ*B and Nrf2 protein expression were decreased. And that it was consistent with previous study that osthole significantly decreased the nuclear factor, Nrf2 protein levels in cervical cancer [37]. Furthermore, Keap 1 protein, an adaptor for the degradation of Nrf2, was lower expressed in HCC mouse models; however, Keap 1 was upregulated with the increase expression of CYP39A1 which was consistent with previous study that it was a negative regulator of Nrf2 protein [38]. Likewise, p-NF-*κ*B and Nrf2 were downregulated after CYP39A1 protein overexpression *in vitro*. Thereby, these all indicated that CYP39A1 might participate in the occurrence and development of the HCC through the NF-*κ*B and Keap1-Nrf2 pathway.

Cholesterol is the starting point for the biosynthesis of steroid hormones, vitamin D, and bile acids, which are primarily metabolized into 24(s)-hydroxycholesterol by CYP46A1 in the brain and 7*α*-hydroxylated by CYP39A1 in the liver, and it is also engaged in the bile acid synthesis pathway. CYP450 plays crucial roles in these intertwined mechanisms of cholesterol homeostasis, such as CYP7A1, 27A1, 46A1, CYP8B1, and CYP39A1, which are cholesterol oxidation products whose expression may be dysregulated in inflammation-related diseases, including cancer [17, 39]. Some of these proteins have been investigated in HCC or cholangiocarcinoma (CCA). For instance, cholesterol elimination-associated genes, such as CYP7A1, 27A1, and CYP8B1, have been investigated in CCA, indicating that only CYP8B1 was associated with CCA overall survival [40]. Another study indicated that hub genes, such as CYP2C8, CYP2C9, and CYP8B1, might be useful as predictive biomarkers for HCC prognosis [41].

To date, CYP39A1, as a novel gene, has been investigated in several other tumors. Currently, it was reported that 24-hydroxycholesterol is involved in the development of pancreatic neuroendocrine tumors [42]. A CYP39A1 single nucleotide polymorphism (rs7761731) was associated with paclitaxel-induced severe neutropenia in patients undergoing chemotherapy for ovarian and endometrial cancer [11]. Furthermore, the promoter of the CYP39A1 gene was hypermethylated in ovarian cancer, resulting in its transcriptional disorder, which caused metabolic disorders of cholesterol and quinoline acid and contributed to the occurrence of ovarian cancer [43]. Moreover, CYP39A1 was expressed at lower levels in malignant melanoma [16] and cholangiocarcinoma [17]. These findings indicated that CYP39A1 was downregulated in several tumors, which was consistent with our study results conducted in HCC.

In the present study, it is worth mentioning that the CYP39A1 mRNA expression difference was not only validated by data from different GSE datasets but also analyzed using data from the TCGA database. Moreover, 14 additional fresh matched liver cases were selected and analyzed by qPCR analysis and Western blotting to validate the expression difference of CYP39A1 mRNA and protein expression. Furthermore, 159 matched noncancerous liver and HCC tissues as well as Akt/c-met-induced HCC mouse models were subjected to IHC analysis to determine the protein expression difference. To analyze the correlation between CYP39A1 expression and clinicopathologic features or HCC survival, protein expression was analyzed by IHC and mRNA expression as analyzed by TCGA database analysis. All the results commonly suggested that CYP39A1 might serve as a prognostic biomarker in HCC occurrence and progression. A similar study also showed that CYP39A1 might be a promising protective-prognostic factor and that altered expression of CYP39A1 might play a useful role in cholangiocarcinoma progression [17].

In addition, to elucidate the role of CYP39A1 in HCC, cell viability and bioinformatic analysis were performed. It is beneficial to take advantage of the DepMap database, which contains genome-wide CRISPR/Cas9 proliferation screening database that can help identify which genes are essential for cell survival and growth. This bioinformatic database provided a simple and effective way for defining and predicting genes that are essential for cell viability [22]. In our previous study, these methods were employed to confirm that RNASET2 was neither a tumor suppressor gene nor an oncogene in gastric carcinoma [21]. Similarly, in this study, by using complete gene knockout techniques, most of the CYP39A1 CERES dependency score of HCC cell lines was >0 with higher score. Therefore, combining the results from cell viability data *in vitro*, CYP39A1 might serve as a tumor suppressor gene in HCC.

Interestingly, in this study, TBA, TBIL, and DBIL were significantly higher in the CYP39A1 low expression group than that in the CYP39A1 high expression group. Recent studies also indicated that CYP39A1 was lower expressed in patients with liver dysfunction. It was reported that CYP39A1 was significantly downregulated in the blood plasma of severe COVID-19 patients with liver dysfunction [44]. Another study revealed that higher hepatic mRNA levels of hepatic CYP39A1 were linked to higher serum cholesterol but protect against steatosis, steatohepatitis, and liver fibrosis in a subset of patients [45]. Furthermore, CYP39A1 could be served as liver toxicity gene markers [46]. Moreover, it was found that CYP39A1 mRNA was increased in models of hepatoprotection mice from cholestasis induced by lithocholic acid (LCA), which toward the formation of less toxic bile acids therefore leading less liver injury [47]. These all recent investigations suggested that CYP39A1 expression levels were associated with the liver function, which was consistent with our observation in the HCC patients. This may suggest the HCC patients with lower expression of CYP39A1 should be prevented from diseases related to the disorder of bile acid metabolism in the clinical treatment. The regulation mechanism between CYP39A1 levels and disorder of bile acid metabolism in HCC needs further investigation.

## 5. Conclusion

In summary, low expression of CYP39A1 was associated with carcinogenesis, tumor differentiation and progression, and poor overall survival of patients with HCC. CYP39A1 might play a role as a tumor suppressor gene and could serve as a potential novel biomarker for HCC.

## Figures and Tables

**Table 1:** Correlation between CYP39A1 expression in tumor cells and clinicopathologic features of HCC.

Clinicopathological features	All cases	CYP39A1 in HCC tissues (%)		*P* value^a^
		Negative	Positive	
Gender				
Male	123	78 (63.41)	45 (36.59)	0.197
Female	36	27 (75.00)	9 (25.00)	
Age at diagnosis (years)				
<51	72	51 (70.83)	21 (29.17)	0.245
≥51	87	54 (62.07)	33 (37.93)	
Size (diameter, cm)				
<5.5	90	57 (63.33)	33 (36.67)	0.411
≥5.5	69	48 (69.57)	21 (30.43)	
Differentiation				
Well	51	24 (47.06)	27 (52.94)	0.001
Moderately and poorly	108	81 (75)	27 (25)	
Nodal metastasis				
*N*0	135	90 (66.67)	45 (33.33)	0.691
*N*1	24	15 (62.50)	9 (37.50)	
Distant metastasis				
*M*0	114	75 (65.79)	39 (34.21)	0.689
*M*1	48	30 (62.50)	18 (37.50)	
TNM stage				
I-II	78	54 (69.23)	24 (30.77)	0.729
III-IV	81	54 (66.67)	27 (33.33)	

^a^Chi-square test.

**Table 2:** Difference of serum biochemical indices between CYP39A1 low expression and CYP39A1 high expression in HCC patients.

Serum biochemical indices	CYP39A1 low expression	CYP39A1 high expression	*P* value^a^
	Mean ± sd	Mean ± sd	
Alanine aminotransferase (ALT)	55.75 ± 74.15	66.21 ± 51.60	0.38
Aspartic transaminase (AST)	51.44 ± 57.93	52.32 ± 43.71	0.93
ALT/AST (ratio)	1.06 ± 0.46	1.28 ± 0.53	0.01
Alkaline phosphatase (ALP)	121.61 ± 101.48	149.39 ± 243.51	0.33
*γ*-Glutamyltransferase (GGT)	112.10 ± 156.95	83.84 ± 64.29	0.23
Total bile acid (TBA)	9.43 ± 7.03	6.46 ± 5.61	0.02
Prealbumin (PA)	69.51 ± 92.25	138.36 ± 115.40	<0.001
Total protein (TP)	65.13 ± 7.98	63.97 ± 8.39	0.42
Albumin (ALB)	38.28 ± 5.71	36.53 ± 6.09	0.09
Globulin (GLB)	26.85 ± 4.23	27.44 ± 4.81	0.45
Ratio of albumin to globulin (A/G)	1.45 ± 0.28	11.57 ± 40.04	0.01
Total bilirubin (TBIL)	25.86 ± 32.48	12.72 ± 5.16	0.01
Direct bilirubin (DBIL)	14.82 ± 28.96	4.89 ± 1.72	0.02
Leucine aminopeptidase (LAP)	68.92 ± 38.34	77.19 ± 37.51	0.25
Glutamate dehydrogenase (GLDH)	7.23 ± 6.69	8.37 ± 9.68	0.44
Fibronectin (Fn)	199.18 ± 40.63	183.90 ± 31.82	0.04
Urea (urea)	5.69 ± 1.38	5.37 ± 2.07	0.28
Creatinine (Cr)	67.37 ± 16.49	78.96 ± 36.44	0.01
Urea nitrogen/creatinine (U/Cr)	0.12 ± 0.16	0.07 ± 0.02	0.06
*β*2-microglobulin (*β*2-m)	2.18 ± 0.68	2.52 ± 1.39	0.06
Total carbon dioxide (TCO2)	26.39 ± 2.97	26.39 ± 2.97	0.19
Uric acid (UA)	294.55 ± 92.49	343.93 ± 121.40	0.01
Total cholesterol (TCh)	3.95 ± 0.93	4.32 ± 1.38	0.10
Triacylglycerol (TG)	1.13 ± 0.63	1.07 ± 0.40	0.62
High-density lipoprotein cholesterol (HDL-Ch)	1.07 ± 0.33	1.09 ± 0.28	0.79
Low-density lipoprotein cholesterin (LDL-Ch)	2.41 ± 0.71	2.67 ± 1.13	0.16
Total/high density lipoprotein cholesterol (T/HDL-Ch)	4.28 ± 3.22	3.98 ± 0.77	0.61
Lipoprotein alpha (LP(*α*))	154.70 ± 156.60	144.93 ± 150.92	0.78

^a^Student's *t*-test.

## Data Availability

The datasets used and analyzed during the current study are available from the corresponding authors on reasonable request.

## Abbreviations

HCC: Hepatocellular carcinoma
CYP450: Cytochrome P450
24OHC: 24 hydroxycholesterol
IHC: Immunohistochemistry
DepMap: The cancer dependency map
CRISPR/Cas 9: Clustered regularly interspaced short palindromic repeats/Cas 9
GEO: Gene Expression Omnibus
TCGA: The Cancer Genome Atlas
qRT-PCR: Quantitative real-time polymerase chain reaction
p-NF-*κ*B: Phosphorylated nuclear factor kappa-B p65
Keap1: Kelch-like ECH-associated protein 1
Nrf2: NF-E2-related factor 2.
